# Association of Adverse Childhood Experience and Attention Deficit Hyperactivity Disorder with depressive symptoms among men who have sex with men in China: moderated mediation effect of resilience

**DOI:** 10.1186/s12889-019-8016-2

**Published:** 2019-12-19

**Authors:** Changmian Ding, Tang Wang, Xiangfan Chen, Jingjing Li, Wei Wang, Danqin Huang, Hong Yan, Shiyue Li

**Affiliations:** 1 School of Health Sciences, Wuhan University, Wuhan, China; 2 Rollins School of Public Health, Emory University, Atlanta, USA; 3 School of Public Health, Xuzhou Medical University, Xuzhou, China

**Keywords:** ACE, ADHD, Resilience, Depressive symptoms, MSM

## Abstract

**Background:**

Adverse childhood experience (ACE), attention deficit hyperactivity disorder (ADHD), and resilience can all contribute to depressive symptoms. However, little is known regarding the complex relationships between these factors and their joint effects on depressive symptoms. This study aimed to explore the underlying mechanism of ACE, ADHD, and resilience on depressive symptoms among men have sex with men (MSM) in China.

**Methods:**

A total of 714 MSM were recruited from gay/bisexual men-serving venues in Wuhan, Changsha, and Nanchang of China. The data was collected using computer-assisted self-interview. The mediated and moderated mediation models were employed to explore the underlying mechanisms between ACE, ADHD, resilience, and depressive symptoms.

**Results:**

Among 714 MSM, 51.4% reported at least one ACE and 13.0% reported three or more. ACE had a direct (β = 1.01, 95% CI: 0.45–1.57) effect on depressive symptoms. ADHD partially mediated the correlation between ACE and depressive symptoms (indirect effect: 0.55; 95% CI: 0.34–0.79). Additionally, the effect of ACE on depressive symptoms was moderated and buffered by resilience (β = −0.09, 95% CI: -0.15 - −0.03).

**Conclusion:**

The findings suggested that, programs and policies that promote resilience and address ADHD might protect Chinese MSM exposed to ACE from depressive symptoms.

## Introduction

### Depressive symptoms among men who have sex with men

Men who have sex with men (MSM), characterized by same-sex behavior, have been identified as a high-risk group for mental disorders, especially depressive symptoms [1, 2]. A study conducted in the United States demonstrated that the prevalence rate of depressive symptoms for MSM was 29.2%, which was much higher than 10.8% in the average adult men [3]. In China, Gao et al. reported that 34.5% of 400 MSM had depressive symptoms in Kunming [4]. The 12-month and lifetime prevalence of major depression were about 2.1 and 2.2 times more compared to general male population [5]. Depression was found to be positively associated with high-risk sexual behaviors and HIV infection in the MSM population [6, 7]. Therefore, reducing depression in MSM population warrants more attention.

### Adverse childhood experience and depressive symptoms among MSM

Adverse childhood experience (ACE) refers to the traumatic experiences occurred under the age of 18, including abuse, neglect and household Challenges [8]. ACE can distort the development of individual and result in increasing risk of poor health outcomes, especially the symptoms of depression [9, 10]. ACEs are common in MSM population. For example, a study in Kenya showed that 70.6% of MSM reported at least one of childhood abuse including one physical abuse and three sexual abuse [11]. Another study conducted in 365 Chinese MSM reported that 12.6% and 10.5% of the respondents experienced psychological and physical abuse under age of 17 [12].

The relationship between ACE and depressive symptoms has been well documented in general population [13–15]. As for MSM, limited studies, for example, Korhonen et al. found that MSM with childhood abuse and recent trauma experienced higher risk of moderate-to-severe depressive symptoms [11]. Hart et al. demonstrated that negative childhood experiences was associated with adult psychological distress among MSM [16]. These studies highlight the need to explore protective factors that extenuate the impact of ACE on depressive symptoms.

### The possible mediated effect of attention deficit hyperactivity disorder (ADHD)

Adverse childhood experience has been identified to relate to attention deficit hyperactivity disorder (ADHD) [13, 17, 18]. It was found that children who experienced socioeconomic hardship, parental divorce, familial mental illness, neighborhood violence, and incarceration had higher risk for ADHD [18]. Individuals who reported more ACE exposures were more likely to experience the symptoms of ADHD [17, 18]. Furthermore, a significant association between ADHD and depressive symptoms has also been found. Studies showed that compared with young people without ADHD, those with ADHD were more likely to experience major depressive disorder [19, 20]. Another study reported that individuals with a history of ADHD had higher level of depressive symptoms throughout adulthood compared to those without ADHD [21]. In addition, a study conducted among 728 adolescents in Netherlands showed that ADHD could increase the risk of depressive symptoms, although this risk reduced over time [22]. Altogether, it is possible that ADHD act as a mediator in the relationship between ACE and depressive symptoms. However, few studies until now have explored this relationship.

### Moderation effect of resilience

Resilience is an indicator of stress coping ability and may reflect the ability adapting or bouncing back from the adverse contexts [23, 24]. Resilience is not a static trait, but rather a dynamic and changing mental process [25], through which individual can cope with adversity without experiencing physically and/or emotionally dysfunctional [23]. Resilience was considered as an important protective factor against the mental disorder following adversity [26]. It has been reported that individuals with higher level of resilience were less likely to have psychiatric disorder symptoms after exposing to significant trauma or adversity [27, 28].

MSM often report ACE exposure and symptoms of depression. Evidence from studies conducted in general population [29], juvenile offenders [30] and postpartum mothers [31] have demonstrated the mitigating effect of resilience in reducing the impact of ACE on depressive symptoms. However, to our knowledge, the possible moderated effect of resilience in MSM population has not been fully investigated.

### Aims and hypotheses

**Fig. 1:**
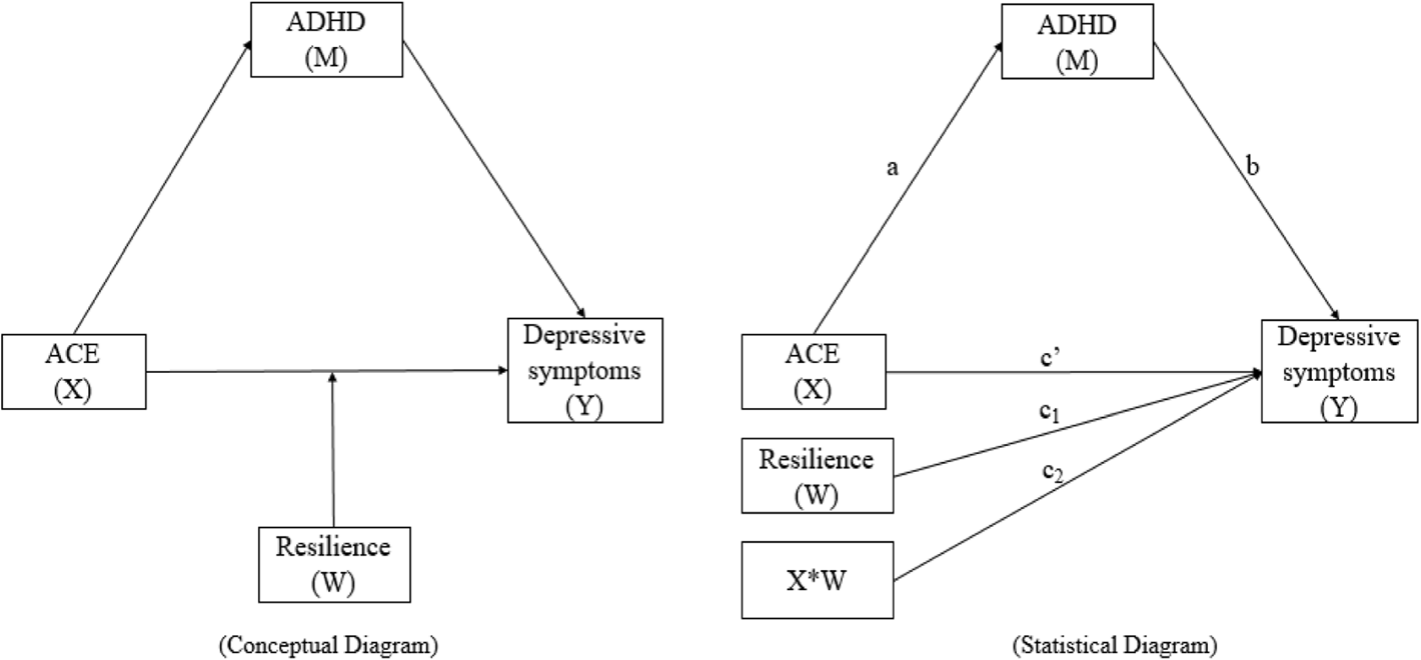
Conceptual (left panel) and statistical diagram (right panel) for the proposed moderated mediation model. Note: (1) X: Adverse childhood experience, ACE; M: Attention deficit hyperactivity disorder, ADHD; W: Resilience; Y: Depressive symptoms;X*W: ACE*Resilience. (2) **a**: direct effect between X and M; **b**: direct effect between M and Y; **c**': direct effect between X and Y; c_1_: direct effect between W and Y; c_2_: direct effect between X*W and Y

Previous studies have reported the associations of ACE, ADHD, and resilience with depressive symptoms [11, 13, 21, 29]. However, whether ADHD mediate the relationship between ACE and depressive symptoms, and whether resilience moderate the direct effect of ACE on depressive symptoms were still little known, especially in MSM population. MSM, as a marginal population, often experience higher level of depressive symptoms than their heterosexual counterpart [4, 5]. It is essential to understand these associations for making interventions to decrease depressive symptoms in MSM. Therefore, in this study, we examined the relationships between ACE, ADHD, resilience and depressive symptoms using data from 714 MSM recruited from three capital cities (Wuhan, Changsha, and Nanchang) in China. As detailed in Fig. 1, three hypotheses were provided: (1) ACE will be positively associated with depressive symptoms; (2) ADHD will be positively associated with depressive symptoms and mediate the relationship between ACE and depressive symptoms; and (3) resilience will moderate and buffer the direct effect of ACE on depressive symptoms.

## Methods

### Participants

Participants for this study were recruited from the gay activity centers and gay-gathering venues, such as gay bars and Karaoke entertainment places (KTV). Eligibility included men aged 16 years or older, self-reported receptive or insertive anal intercourse or oral sex with another man in their lifetime, and agreed to participate in this study.

### Procedure

Data of this study was derived from the baseline survey of a MSM cohort study in China. The Medical Ethics Committee at Wuhan University of China reviewed and approved for this project.

Participants were recruited through various ways, such as routine HIV testing services, outreach activities, peer recommendation, and so on, in three capital cities of China: Wuhan, Nanchang and Changsha, from September 2017 to January 2018. All recruitment activities were supported by gay organizations (e.g. Tongxing of Wuhan). Every survey site was assigned to two well-trained graduate students, which would be responsible for recruiting participants and assisting the survey. This is an anonymous self-administered questionnaire survey. After completing informed consent, electronic questionnaires were provided to participants through the tablets (e.g. iPad). Participants completed the questionnaires independently in a quiet room in order to protect the individual privacy. A face-to-face assistance could be provided if the participant had any questions about the questionnaire. It took approximately 30 min for most participants to complete the questionnaire. Participants received a reward of 50 RMB (approximately US$8) after completing the questionnaire. Among 800 eligible MSM, 749 of them completed and submitted the questionnaires (response rate: 93.63%). A sample of 714 was used to analyze after excluding the individuals with incomplete data for key variables.

### Measurement

#### Demographics characteristic

Information on age, residence, education, marital status, students status, employment status, monthly income, and sexual orientation was obtained. Residence was divided into urban and rural. Education was categorized as high school or lower and college or higher. Marital status was classified as unmarried, married, and divorced. Students status was divided into yes and no. Employment status was categorized as full-time employment, part-time employment, and others. Monthly income (in RMB) was divided into: ≤1000, 1001–3000, 3001–6000, >6000. Sexual orientation was classified as gay, bisexual, and undecided.

#### Adverse childhood experience (ACE)

ACE was evaluated using ACE questionnaire developed in Kaiser-CDC study [8, 32]. Participant reported 10 questions related to their three dimensions of experiences before age 18, including abuse (emotional, physical and sexual), neglect (emotional and physical) and household challenges (mother’s violence, household substance abuse, household mental illness, parental separation or divorce, and household member incarcerated). Example item was “Did a parent, stepparent, or other adult living in your home swore at you, put you down, or acted in a way that made you afraid that you might be physically hurt?”. Each item consists of three responses: “yes”, “no” and “refuse”. Responses of “yes” were considered that participants had the adverse childhood experience and scored as “1”. Total score was calculated by adding up all responses with higher scores reflecting more adverse childhood experiences. The instrument was reported to have a good reliability and validity in Chinese population [33–35]. The Cronbach’s Alpha in our sample was 0.77.

#### Attention deficit hyperactivity disorder (ADHD)

Adult ADHD Self-Report Scale (ASRS-v1.1) [36, 37] was applied to measure ADHD of the participants. It is an 18-items instrument with 5-piont rating (0 = never, 1 = rarely, 2 = sometimes, 3 = often, 4 = very often). The scale consisted of two subscales, inattention (9 items, e.g. How often do you have trouble wrapping up the final details of a project, once the challenging parts have been done?) and hyperactivity-impulsivity (9 items, e.g. How often do you fidget or squirm with your hands or feet when you have to sit down for a long time?). The total score ranges from 0 to 72, with higher scores indicating higher level of ADHD. The instrument has been used in Chinese sample, and showed a good reliability [38]. The Cronbach’s Alpha for our sample were 0.92 for total scale, 0.85 for inattention domain, and 0.86 for hyperactivity-impulsivity domain.

#### Resilience

Connor-Davidson Resilience Scale-10 items (CD-RISC-10) [39] was used to assess resilience. The items of the scale included “I am able to adapt to change”, “Coping stress can strengthen me”, “I can achieve goals despite obstacles”, and so on. The items are scored on a 5-point rating scale from 0 (never) to 4 (always), with total score of 0–40. Higher scores indicated greater resilience. The scale was reported to have a good reliability and validity in Chinese population [40, 41]. The Cronbach’s Alpha in present sample was 0.95.

#### Depressive symptoms

The Centers for Epidemiological Studies Depression Scale (CESD) [42] was applied to evaluate depressive symptoms experienced in the past 7 days. The CESD is a 20-item, including four dimensions: depressed affect (8 items, e.g. I feel depressed.), positive affect (4 reverse items, e.g. I’m very happy.), somatic and retarded activity (6 items, e.g. I have a hard time focusing on doing things.), and interpersonal problems (2 items, e.g. I think others hate me.). It is a four-point rating scale ranging from 0 (never) to 3 (always). The total score was 0–60, and higher scores indicated higher levels of depressive symptoms. The CESD has been widely used in Chinese population, and showed adaptable reliability and validity [43, 44], as well as the sample of MSM [45]. In this study, the Cronbach’s Alpha of CESD was 0.87. As for the four subscales, the Cronbach’s Alpha ranged from 0.76 to 0.87.

### Statistical analysis

Descriptive analysis was performed to describe sociodemographic characteristics. Confirmatory factor analysis (CFA) was conducted to evaluate the construct validity of the scales using Mplus 7.0. The fit indices of χ2/df (< 3), Root Mean Square Error of Approximation (RMSEA; < 0.08), Comparative Fit Index (CFI; > 0.90), Tucker Lewis index (TLI; > 0.90) and Standardized Root Mean Square Residual (SRMR; < 0.08)/Weighted Root Mean Square Residual (WRMR; < 1.00) were used to assess the fitness of the model [46]. In addition, convergent validity was assessed by calculating the value of Average Variance Extracted (AVE; > 0.5) and Composite Reliability (CR; > 0.7) [46]. And discrimination validity was assessed by comparing the chi-square differences value (Δλ^2^; > 3.84, > 6.64, or > 10.83) between unconstrained model (CFA for two different dimensions of a scale) and constrained models (CFA after merging the two different dimensions into one dimension) [46, 47].

**Fig. 2:**
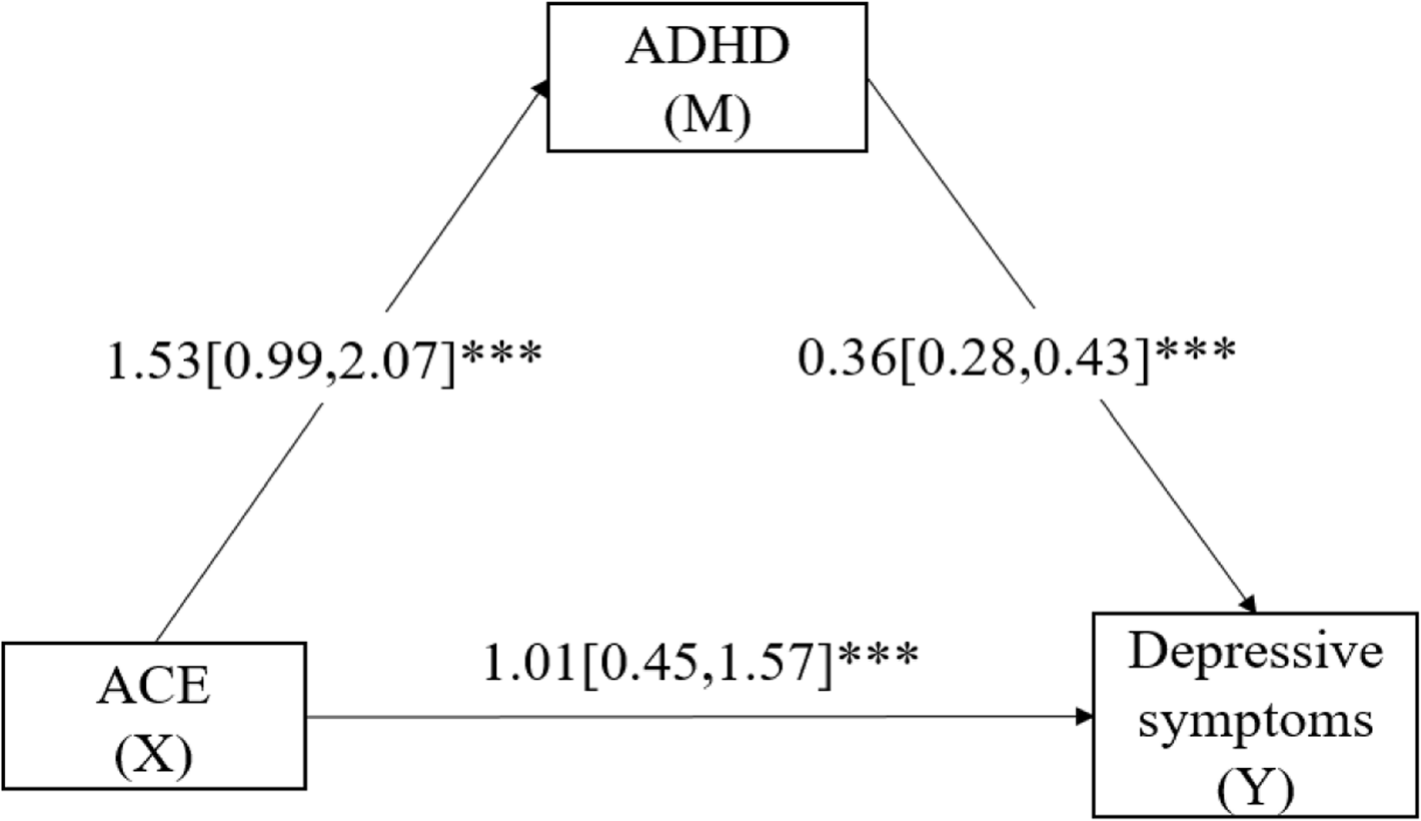
(Model 4). Mediation modeling analysis of the relationship among ACE, ADHD and depressive symptoms among MSM (*N* = 714). Notes: (1) Covariates controlled in the modeling analysis were: age, residence, marital status, education, student, employment status, monthly income, and sexual orientation. (2) ACE: Adverse childhood experience; ADHD: Attention deficit hyperactivity disorder. (3) *** *p* < 0.001

**Fig. 3:**
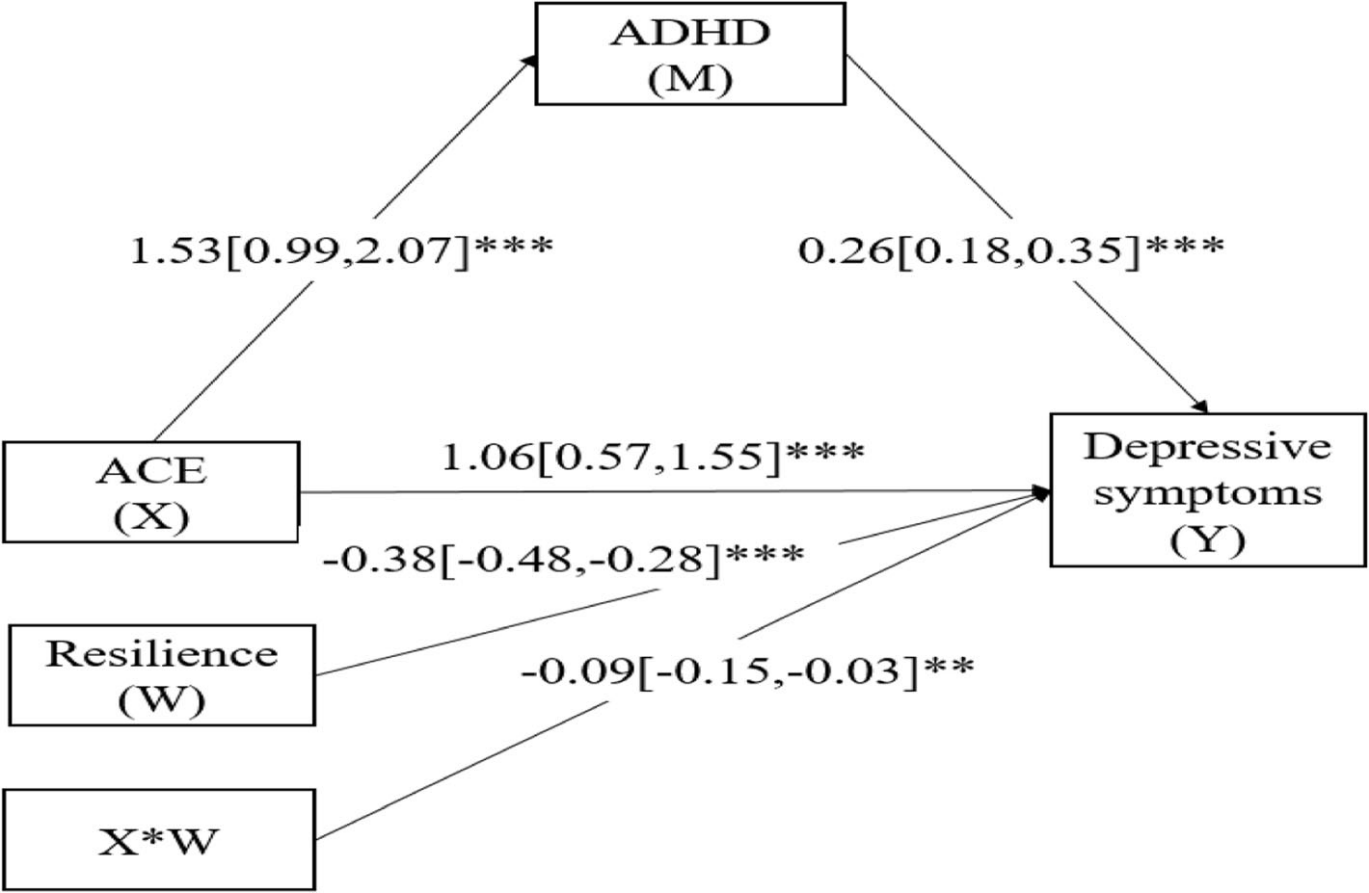
(Model 5). Moderated mediation modeling analysis of the complex relationship among ACE, ADHD, resilience, and depressive symptoms of MSM (*N* = 714). Notes: (1) Covariates controlled in the modeling analysis were age, residence, marital status, education, student, employment status, monthly income and sexual orientation. (2) ACE: Adverse childhood experience; ADHD: Attention deficit hyperactivity disorder. (3) **p < 0.01, ***p < 0.001

Pearson correlation analysis was conducted to analyze the bivariate correlations between ACE, ADHD, resilience, and depressive symptoms. The PROCESS macro for SPSS [48] was used for the mediation and moderated mediation analysis. At first, the mediated effect of ADHD between ACE and depressive symptoms was examined by model 4 (see Fig. 2). Then, model 5 was used to test the moderated mediation effect of resilience, that was, whether resilience moderated the direct effect of ACE on depressive symptoms or not (see Fig. 3). Finally, we plotted the conditional effects of resilience at low (1 standard deviation [SD] below the mean value of resilience scale), moderate (the mean value of resilience scale) and high (1SD above the mean of resilience scale) levels. In addition, covariates (age, education, residence, marital status, student, employment status, monthly incomes and sexual orientation) were controlled in all model analysis and all study variables were mean-centered prior to the analysis. All statistical analysis excluded CFA was conducted with the software of SPSS 22.0.

## Results

### Sample characteristics

A total of 714 participants was recruited in this study, 60.5% of them were from urban areas, with a mean age of 27.1 (SD = 8.5) years old. Around three-quarters (74.8%) of the sample had college or higher-level education, 27.5% were students, and 58.8% had a full-time job. Of the full sample, 84.3% reported never married and approximately half of participants had monthly income less than 3000 RMB (approximately USD $480), and 73.0% of the participants self-reported as gay men (see Table 1).

**Table 1:** Characteristics of the study sample (*n* = 714)

variables	N	%
Age (in years)		
Range	16–62	
Mean (SD)	27.1	8.5
Residence		
Urban	432	60.5
Rural	282	39.5
Education		
High school or lower	180	25.3
College or higher	534	74.8
Student		
Yes	196	27.5
No	518	72.5
Employment status		
Full-time job	420	58.8
Part-time job/others	294	41.2
Marital status		
Unmarried	602	84.3
Married/Divorced	112	15.7
Monthly income		
≤ 1000	92	12.9
1001–3000	261	36.6
3001–6000	228	31.9
>6000	133	18.6
Sexual orientation		
Gay	521	73.0
Bisexual/undecided	193	27.0

### Evidence for validity

Table 2 displayed the results of the CFA and convergent validity analysis for the four scales. Results from CFA with the scale of ACE (χ^2^/df = 2.91, RMSEA = 0.036, CFI = 0.977, TLI = 0.967, WRMR = 0.930), ADHD (χ^2^/df = 4.86, RMSEA = 0.074, CFI = 0.907, TLI = 0.892, SRMR = 0.044), resilience (χ^2^/df = 5.11, RMSEA = 0.076, CFI = 0.961, TLI = 0.947, SRMR = 0.042), and depressive symptoms (χ^2^/df = 2.80, RMSEA = 0.050, CFI = 0.930, TLI = 0.919, SRMR = 0.045) showed acceptable fit. All values of CR for ACE scale met the cut-off criteria, ranging from 0.72 to 0.85. The AVE value of ACE domains were acceptable, except for household challenges (AVE = 0.38). Although ADHD acquired lower value of AVE, the CR values of both domains reached 0.86, which obviously higher than acceptable level 0.7. The resilience scale showed good convergent validity, the values of CR and AVE both met the cut-off criteria, reaching 0.94, 0.62, respectively. As for the scale of depressive symptoms, although the domain of somatic and restarted activity presented limited AVE values, the AVE values of other domains were around 0.5. And all values of CR were over the recommended threshold of 0.70, ranging from 0.78 to 0.87. Above results indicated that the construct validity and convergent validity of four scales were acceptable.

**Table 2:** Analysis of the construct validity and convergent validity of scales

Scales	Domains	Items	λ	CR	AVE
ACE					
	Abuse	3	0.34–0.95	0.81	0.62
	Neglect	2	0.78–0.94	0.85	0.74
	Household challenges	5	0.39–1.05	0.72	0.38
	χ^2^/df = 2.91, RMSEA = 0.036, CFI = 0.977, TLI = 0.967, WRMR = 0.930				
ADHD		18	0.40–0.79	0.92	0.41
	Inattention	9	0.40–0.79	0.86	0.41
	Hyperactivity	9	0.54–0.72	0.86	0.40
	χ^2^/df = 4.86, RMSEA = 0.074, CFI = 0.907, TLI = 0.892, SRMR = 0.044				
Resilience					
	Resilience	10	0.68–0.85	0.94	0.62
	χ^2^/df = 5.11, RMSEA = 0.076, CFI = 0.961, TLI = 0.947, SRMR = 0.042				
Depressive symptoms					
	Depressed affect	8	0.55–0.77	0.87	0.46
	Positive affect	4	0.53–0.77	0.79	0.48
	Somatic and restarted activity	6	0.47–0.76	0.78	0.37
	Interpersonal	2	0.77–0.83	0.78	0.64
	χ^2^/df = 2.80, RMSEA = 0.050, CFI = 0.930, TLI = 0.919, SRMR = 0.045				

Note: λ: Factor loading value; AVE: Average Variance Extracted; CR: Composite Reliability; ACE: Adverse childhood experience; ADHD: Attention deficit hyperactivity disorder; RMSEA: Root Mean Square Error of Approximation; CFI: Comparative Fit Index; TLI: Tucker Lewis index; SRMR: Standardized Root Mean Square Residual; WRMR: Weighted Root Mean Square Residual

Table 3 displayed the results of discrimination validity for the scales. Results showed that **Δχ**^**2**^ between unconstrained model (Model A) and constrained model (Model B) for the scale of ACE, ADHD, depressive symptoms were 10.97–11.21, 76.50, 13.17–650.05, respectively. The values were significantly higher than the critical value 10.83 (*P* < 0.001). Those results indicated that the scale of ACE, ADHD, depressive symptoms have a good discrimination validity.

**Table 3:** Analysis of the discriminant validity of scales

Scale	Paired-dimensions	Model A		Model B		Model B-Model A	
		χ^2^	df	χ^2^	df	Δχ^2^	Δdf
ACE							
Abuse-Neglect		13.17	4	24.38	5	11.21^***^	1
Abuse-Household challenge		37.25	19	49.17	20	11.92^***^	1
Neglect-Household challenge		34.63	13	45.60	14	10.97^***^	1
ADHD							
Inattention-Hyperactivity		641.57	132	718.07	133	76.50^***^	1
Depressive symptoms							
Depressed affect-Positive affect		169.47	53	766.59	54	597.59^***^	1
Depressed affect-Somatic and restarted activity		252.23	76	265.40	77	13.17^***^	1
Depressed affect- Interpersonal		160.46	34	210.68	35	50.46^***^	1
Positive affect- Somatic and restarted activity		79.13	34	729.18	25	650.05^***^	1
Positive affect- Interpersonal		14.36	8	312.29	9	297.93^***^	1
Somatic and restarted activity- Interpersonal		52.72	19	88.94	20	36.22^***^	1

Note:(1) Model A was unconstrained model; Model B was constrained model; (2) ^*^Δχ^2^ > 3.84, P < 0.05; ^**^Δχ^2^ > 6.64, *P* < 0.01; ^***^Δχ^2^ > 10.83, P < 0.001; (3) ACE: Adverse childhood experience; ADHD: Attention deficit hyperactivity disorder; (4) The discriminant validity of resilience scale was not analyzed because only one dimension was included

### Bivariate correlations

Table 4 displayed the mean, standard deviation and correlations of ACE, ADHD, resilience, and depressive symptoms. The score of ACE was 1.04 (SD = 1.43) ranging from 0 to 9. Among 714 MSM, 367 (51.4%) reported at least one of ACE exposures and 93 (13.0%) reported three or more exposures. The mean scores of ADHD, resilience and depressive symptoms were 21.86 (SD = 11.34), 26.78 (SD = 8.45) and 17.64 (SD = 10.49), respectively.

**Table 4:** Correlation between ACE, ADHD, resilience, and depressive symptoms among MSM (N = 714)

Variable	Mean	SD	ACE	ADHD	Resilience	Depressive symptoms
1. ACEs	1.04	1.43	1	0.19^***^	−0.05	0.22^***^
2. ADHD	21.86	11.34		1	−0.31^***^	0.39^***^
3. Resilience	26.78	8.45			1	−0.39^***^
4. Depressive symptoms	17.64	10.49				1

Note: (1) ACE: Adverse childhood experience; ADHD: Attention deficit hyperactivity disorder. (2)***p < 0.001

Results in Table 4 indicated that ACE was positively associated with ADHD (r = 0.19, *P* < 0.001) and depressive symptoms (r = 0.22, *P* < 0.001) among MSM. Resilience was significantly associated with ADHD (r = − 0.31, *P* < 0.001) and depressive symptoms (r = − 0.39, *P* < 0.001) except ACE (r = − 0.05, *P* = 0.20). In addition, ADHD was found to be positively associated with depressive symptoms (r = 0.39, *P* < 0.001).

### Mediation modeling analysis

Results in Fig. 2 displayed that ACE was significantly and positively associated with depressive symptoms (β = 1.01, 95% CI: 0.45–1.57). Furthermore, ACE was also found to be positively associated with ADHD (β = 1.53, 95% CI: 0.99–2.07), which in turn was significantly predictive of depressive symptoms (β = 0.36, 95% CI: 0.28–0.43). Results demonstrated that ADHD partially mediated the relationship between ACE and depressive symptoms for MSM (indirect effect = 0.55, bootstrap 95%CI: 0.34–0.79, accounting for 35.25% of the total effect).

### Moderated mediation analysis

Moderated mediation analysis in Fig. 3 showed that all paths in the model were significantly remarkable (*P* < 0.05), including the interaction effect of ACE and resilience (β = − 0.09, 95% CI: − 0.15 - − 0.03). Results indicated that resilience was significantly and negatively moderated the relation between ACE and depressive symptoms for MSM.

### Conditional direct effect of moderated mediation analysis

**Fig. 4:**
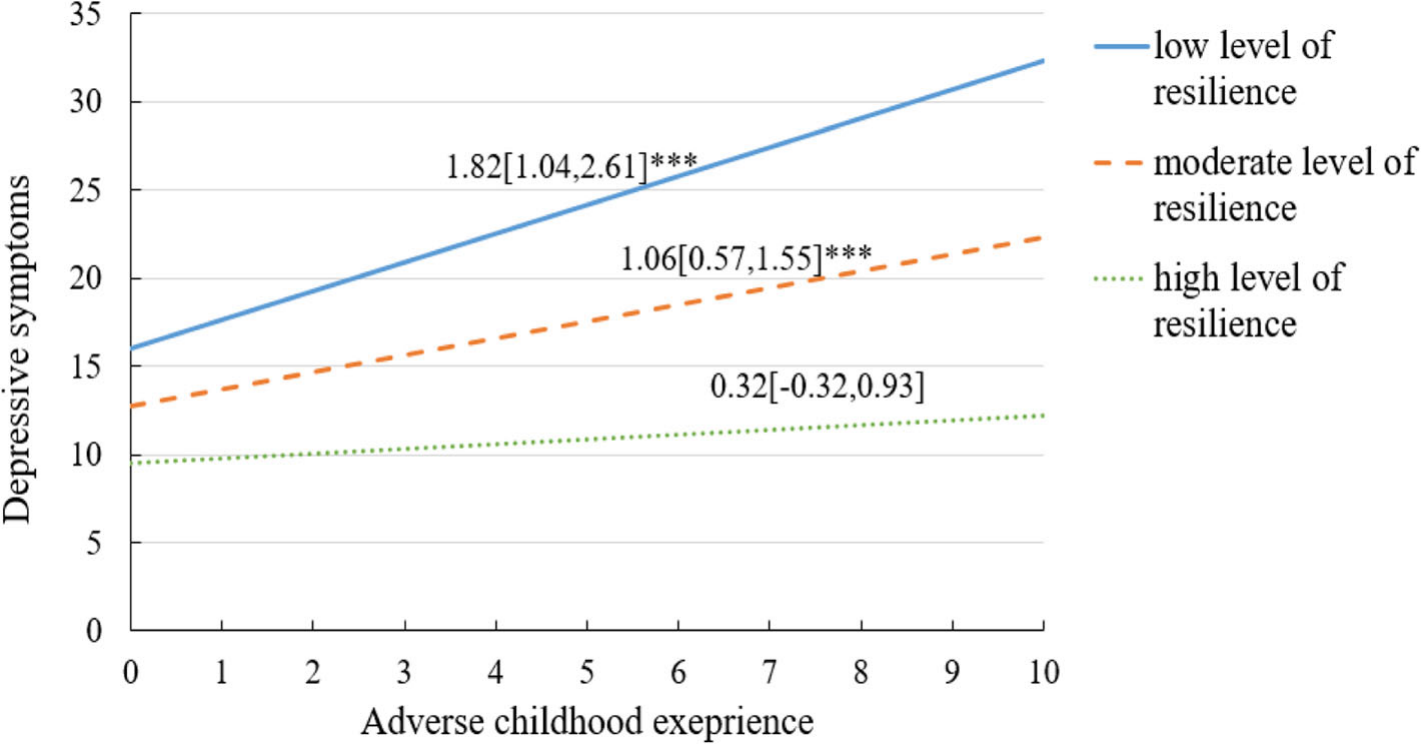
The moderating role of resilience in the relation between adverse childhood experience and depressive symptoms among MSM (N = 714). Notes: (1) Covariates controlled in the modeling analysis were age, residence, marital status, education, student, employment status, monthly income and sexual orientation. (2) ***p < 0.001

The conditional effects of resilience at low level (i.e., 1 SD below the mean), moderate level (i.e., mean) and high level (i.e., 1 SD above the mean) were depicted in Fig. 4, respectively. Results illustrated that among MSM with low level of resilience, higher ACE score were associated with higher risk of depressive symptoms (β = 1.82, 95%CI: 1.04–2.61). Furthermore, the effect at the moderate level of resilience was also significant (β = 1.06, 95%CI: 0.57–1.55). However, for those MSM with high levels of resilience, the relationship between ACE and depressive symptoms was non-significant (β = 0.32, 95%CI: − 0.32 - 0.93).

## Discussion

This study firstly examined construct validity as well as convergent validity and discrimination validity of the ACE, ADHD, resilience, and depressive symptoms scales in the sample of MSM. Results indicated that the validity of four scales were all acceptable. Secondly, the mediating role of ADHD between ACE and depressive symptoms, and the moderating effect of resilience among ACE, ADHD and depressive symptoms were explored, using a moderated mediation model. The findings demonstrated that ACE, ADHD and resilience were significantly associated with depressive symptoms, however, the underlying effects of these factors on depressive symptoms were different. ADHD partially mediated the relationship between ACE and depressive symptoms. Moreover, the direct effect of the mediation model was moderated by resilience, that the direct effect of ACE on depressive symptoms decreased as resilience increased.

MSM in our study reported higher scores of ACE, with over half of participants reporting one or more ACE exposures, compared to general male population [13, 49]. Our results showed a strong positive association of ACE with depressive symptoms, which was in accordance with that of previous studies [13–15]. The finding could be explained by the stress sensitization theory [50]. This theory suggests that the threshold of depressive reactions can be reduced by ACE, which will contribute to significant depressive reactions of individual even exposed to mild stress. MSM usually experienced various kinds of ACE prior to the age of 18 years [11, 12]. In addition, MSM, a marginalized and isolated population, often confronted with various stressors or challenges such as stigma [51, 52]. Therefore, MSM often have higher prevalence of depressive symptoms.

As hypothesized, we found ACE was positively associated with ADHD, which in turn escalated the symptoms of depression. The finding possibly revealed that ADHD served as an important mediating mechanism concerning how ACE indirectly affected depressive symptoms. To our knowledge, this study was the first to examine the underlying mediated mechanism of ADHD in the relation between ACE and depressive symptoms, as previous studies only examined the relationship among ACE, ADHD and depressive symptoms separately [13, 22]. The mediating effect of ADHD might be attribute to the following reasons. ACE exposures might cause changes in brain structures and functioning related to ADHD, which resulted in the development of ADHD [53–55]. However, individual with ADHD often have poor academic achievement, unstable professional career, more fiscal problems and unsatisfied interpersonal relation [56], due to the difficulties in thinking, concentration, organizational skill and so on [57]. Therefore, they were more likely to experience to depressive symptoms. The finding suggested that the diagnosis and treatment of ADHD might be beneficial to the intervention work on depressive symptoms among MSM population.

Additionally, resilience moderated and buffered the effect size of the correlation between ACE and depressive symptoms (direct effect). We found that as resilience increased, the association of ACE with depressive symptoms weakened and became non-significant when resilience reached to the high-level. This finding aligns with one study conducted in the United States [29], which suggested that resilience might reduce the tendency for developing depressive symptoms towards similar levels of childhood adversity. Similarly, a survey among 429 juvenile adolescents in the US [30] also reported the moderated effect of internal resilience in the relation of ACE to psychological distress. Our findings could be explained by the characteristics of resilience. Resilience was defined as an adaptable ability that enable one to thrive following adversity [24]. Individual with high resilience often keeps a sensitive and positive attitude towards adversity, which can prompt them to bounce back from the disadvantaged environments [23, 58]. Therefore, resilience could serve as a protective factor and mitigate the impact of ACE on depressive symptoms, which indicates building up resilience might help to ease depressive symptoms among MSM.

### Limitations

There are some limitations in this study. First, due to the cross-sectional nature of the data, the causality between variables cannot be established. Also, temporality of some variables, such as ACE, ADHD and resilience, could not be captured. Therefore, our findings need to be validated in longitudinal study in the future. Next, data were self-reported, thus, there could be self-report bias in this study. Furthermore, the participants recruited in current study were mainly from three capital cities of China, which may not represent all MSM in China, such as MSM from remote or rural areas. Finally, some potentially important protective factors such as family and community support were not assessed. We controlled for some sociodemographic variables in our analyses, but residual confounding may still be present.

## Conclusion

This study contributes to the literature by showing that ADHD partially mediates the relation between ACE and depressive symptoms, and resilience buffers the direct effect of ACE on depressive symptoms. MSM with higher ACE scores, especially those who have more symptoms of ADHD and present low and moderate levels of resilience were more likely to experience depressive symptoms. Chinese public health practitioners aiming to improve MSM’s mental health should consider the effect of ACE on depressive symptoms and the role of ADHD and resilience in the relation.

## Data Availability

The data used in this study are available from the corresponding authors on reasonable request.

## Abbreviations

ACE: Adverse childhood experience
ADHD: attention deficit hyperactivity disorder
AVE: The Average Variance Extracted
CFA: Confirmatory factor analysis
CFI: Comparative Fit Index
CI: Confidence interval
CR: Component reliability
HIV: human immunodeficiency virus
KTV: Karaoke entertainment places
MSM: men who have sex with men
RMSEA: Root Mean Square Error of Approximation
SRMR: Standardized Root Mean Square Residual
TLI: Tucker Lewis index
WRMR: Weighted Root Mean Square Residual

## References

[1] Sivasubramanian M, Mimiaga MJ, Mayer KH, Anand VR, Johnson CV, Prabhugate P, Safren SA (2011). Suicidality, clinical depression, and anxiety disorders are highly prevalent in men who have sex with men in Mumbai, India: findings from a community-recruited sample. Psychol Health Med.

[2] Stoloff K, Joska JA, Feast D, De Swardt G, Hugo J, Struthers H, McIntyre J, Rebe K (2013). A description of common mental disorders in men who have sex with men (MSM) referred for assessment and intervention at an MSM clinic in Cape Town, South Africa. AIDS Behav.

[3] Mills TC, Paul J, Stall R, Pollack L, Canchola J, Chang YJ, Moskowitz JT, Catania JA (2004). Distress and depression in men who have sex with men: the urban Men's health study. Am J Psychiatry.

[4] Gao XJZR, Li YF, Ma YL, Chen YL, Ma J, Li ZQ, Jia MH (2018). Depression and related influential factors among men who have sex with men in Kunming. Chin J AIDS STD.

[5] Yu LZ, Jiang C, Na J, Li N, Diao WL, Gu Y, Zhao L, Zou Y, Chen Y, Liu L (2013). Elevated 12-Month and lifetime prevalence and comorbidity rates of mood, anxiety, and alcohol use disorders in Chinese men who have sex with men. PLoS One.

[6] Mimiaga MJ, O'Cleirigh C, Biello KB, Robertson AM, Safren SA, Coates TJ, Koblin BA, Chesney MA, Donnell DJ, Stall RD (2015). The effect of psychosocial syndemic production on 4-year HIV incidence and risk behavior in a large cohort of sexually active men who have sex with men. J Acquir Immune Defic Syndr (1999).

[7] Ahaneku H, Ross MW, Nyoni JE, Selwyn B, Troisi C, Mbwambo J, Adeboye A, McCurdy S (2016). Depression and HIV risk among men who have sex with men in Tanzania. Aids Care-Psychological Socio-Medical Aspects Aids/Hiv.

[8] Centers for Disease Control and Prevention: adverse childhood experiences (ACE) study. Available from: http://www.cdc.gov/ace/about.htm. Accessed 12 Apr 2019.

[9] Hughes K, Bellis MA, Hardcastle KA, Sethi D, Butchart A, Mikton C, Jones L, Dunne MP (2017). The effect of multiple adverse childhood experiences on health: a systematic review and meta-analysis. Lancet Public Health.

[10] Brockie TN, Dana-Sacco G, Wallen GR, Wilcox HC, Campbell JC (2015). The relationship of adverse childhood experiences to PTSD, depression, poly-drug use and suicide attempt in reservation-based native American adolescents and young adults. Am J Community Psychol.

[11] Korhonen C, Kimani M, Wahome E, Otieno F, Okall D, Bailey RC, Harper GW, Lorway RR, Doshi M, Mathenge J (2018). Depressive symptoms and problematic alcohol and other substance use in 1476 gay, bisexual, and other MSM at three research sites in Kenya. AIDS (London).

[12] Zhu Y, Liu J, Chen Y, Zhang R, Qu B (2018). The relation between mental health, homosexual stigma, childhood abuse, community engagement, and unprotected anal intercourse among MSM in China. Sci Rep.

[13] Windle M, Haardorfer R, Getachew B, Shah J, Payne J, Pillai D, Berg CJ (2018). A multivariate analysis of adverse childhood experiences and health behaviors and outcomes among college students. J Am Coll Heal.

[14] Salokangas RKR, From T, Luutonen S, Hietala J (2018). Adverse childhood experiences leads to perceived negative attitude of others and the effect of adverse childhood experiences on depression in adulthood is mediated via negative attitude of others. Eur Psychiatry.

[15] Cheong EV, Sinnott C, Dahly D, Kearney PM (2017). Adverse childhood experiences (ACEs) and later-life depression: perceived social support as a potential protective factor. BMJ Open.

[16] Hart TA, Noor SW, Vernon JRG, Kidwai A, Roberts K, Myers T, Calzavara L (2018). Childhood maltreatment, bullying victimization, and psychological distress among gay and bisexual men. J Sex Res.

[17] Jimenez ME, Wade R, Schwartz-Soicher O, Lin Y, Reichman NE (2017). Adverse childhood experiences and ADHD diagnosis at age 9 years in a National Urban Sample. Acad Pediatr.

[18] Brown NM, Brown SN, Briggs RD, Germán M, Belamarich PF, Oyeku SO (2017). Associations between adverse childhood experiences and ADHD diagnosis and severity. Acad Pediatr.

[19] Biederman J, Ball SW, Monuteaux MC, Mick E, Spencer TJ, McCreary M, Cote M, Faraone SV (2008). New insights into the comorbidity between ADHD and major depression in adolescent and young adult females. J Am Acad Child Adolesc Psychiatry.

[20] Chronis-Tuscano A, Molina BS, Pelham WE, Applegate B, Dahlke A, Overmyer M, Lahey BB (2010). Very early predictors of adolescent depression and suicide attempts in children with attention-deficit/hyperactivity disorder. Arch Gen Psychiatry.

[21] Meinzer MC, Pettit JW, Waxmonsky JG, Gnagy E, Molina BS, Pelham WE (2016). Does childhood attention-deficit/hyperactivity disorder (ADHD) predict levels of depressive symptoms during emerging adulthood?. J Abnorm Child Psychol.

[22] Roy A, Hartman CA, Veenstra R, Oldehinkel AJ (2015). Peer dislike and victimisation in pathways from ADHD symptoms to depression. Eur Child Adolesc Psychiatry.

[23] Tugade MM, Fredrickson BL, Barrett LF (2004). Psychological resilience and positive emotional granularity: examining the benefits of positive emotions on coping and health. J Pers.

[24] Connor KM, Davidson JR (2003). Development of a new resilience scale: the Connor-Davidson resilience scale (CD-RISC). Depress Anxiety.

[25] Luthar SS, Cicchetti D, Becker B (2000). The construct of resilience: a critical evaluation and guidelines for future work. Child Dev.

[26] Rutter M (1985). Resilience in the face of adversity. Protective factors and resistance to psychiatric disorder. Br J Psychiatry.

[27] Alim TN, Feder A, Graves RE, Wang Y, Weaver J, Westphal M, Alonso A, Aigbogun NU, Smith BW, Doucette JT (2008). Trauma, resilience, and recovery in a high-risk African-American population. Am J Psychiatry.

[28] Collishaw S, Pickles A, Messer J, Rutter M, Shearer C, Maughan B (2007). Resilience to adult psychopathology following childhood maltreatment: evidence from a community sample. Child Abuse Negl.

[29] Youssef NA, Belew D, Hao G, Wang X, Treiber FA, Stefanek M, Yassa M, Boswell E, McCall WV, Su S (2017). Racial/ethnic differences in the association of childhood adversities with depression and the role of resilience. J Affect Disord.

[30] Clements-Nolle K, Waddington R (2019). Adverse childhood experiences and psychological distress in juvenile offenders: the protective influence of resilience and youth assets. J Adolesct Health.

[31] Sexton MB, Hamilton L, McGinnis EW, Rosenblum KL, Muzik M (2015). The roles of resilience and childhood trauma history: main and moderating effects on postpartum maternal mental health and functioning. J Affect Disord.

[32] Felitti VJ, Anda RF, Nordenberg D, Williamson DF, Spitz AM, Edwards V, Koss MP, Marks JS (1998). Relationship of childhood abuse and household dysfunction to many of the leading causes of death in adults. The adverse childhood experiences (ACE) study. Am J Prev Med.

[33] Ding YY, Lin HJ, Zhou L, Yan HM, He N (2014). Adverse childhood experiences and interaction with methamphetamine use frequency in the risk of methamphetamine-associated psychosis. Drug Alcohol Depend.

[34] Liu Z, Yang Y, Shi Z, Liu J, Wang Y (2016). The risk of male adult alcohol dependence: the role of the adverse childhood experiences and ecological executive function. Compr Psychiatry.

[35] Fung HW, Ross CA, Yu CKC, Lau EKL (2019). Adverse childhood experiences and dissociation among Hong Kong mental health service users. J Trauma Dissociation.

[36] Kessler RC, Adler L, Ames M, Demler O, Faraone S, Hiripi E, Howes MJ, Jin R, Secnik K, Spencer T (2005). The World Health Organization adult ADHD self-report scale (ASRS): a short screening scale for use in the general population. Psychol Med.

[37] Merikanto I, Kuula L, Makkonen T, Halonen R, Lahti J, Heinonen K, Raikkonen K, Pesonen AK (2019). ADHD symptoms are associated with decreased activity of fast sleep spindles and poorer procedural overnight learning during adolescence. Neurobiol Learn Mem.

[38] Tong L, Shi HJ, Zhang Z, Yuan Y, Xia ZJ, Jiang XX, Xiong X (2016). Mediating effect of anxiety and depression on the relationship between attention-deficit/hyperactivity disorder symptoms and smoking/drinking. Sci Rep.

[39] Campbell-Sills L, Stein MB (2007). Psychometric analysis and refinement of the Connor-davidson resilience scale (CD-RISC): validation of a 10-item measure of resilience. J Trauma Stress.

[40] Wang L, Shi Z, Zhang Y, Zhang Z (2010). Psychometric properties of the 10-item Connor-Davidson resilience scale in Chinese earthquake victims. Psychiatry Clin Neurosci.

[41] Zhang M, Zhang JM, Zhang F, Zhang L, Feng DJ (2018). Prevalence of psychological distress and the effects of resilience and perceived social support among Chinese college students: does gender make a difference?. Psychiatry Res.

[42] Radloff LS (1977). The CES-D scale: a self-report depression scale for research in the general population. Appl Psychol Meas.

[43] Jie Zhang ZW, Fang G, Li J, Han B, Chen Z (2010). Development of the Chinese age norms of CESD in urban area. Chin Ment Health J.

[44] Wang W, Xiao CC, Yao X, Yang YM, Yan H, Li SY (2018). Psychosocial health and suicidal ideation among people living with HIV/AIDS: a cross-sectional study in Nanjing, China. PLoS One.

[45] Yan H, Li XY, Li JJ, Wang W, Yang YM, Yao X, Yang NX, Li SY (2019). Association between perceived HIV stigma, social support, resilience, self-esteem, and depressive symptoms among HIV-positive men who have sex with men (MSM) in Nanjing, China. Aids Care-Psychological Socio-Medical Aspects Aids/Hiv.

[46] Hair JF, Black WC, Babin BJ, Anderson RE (2010). Multivariate data analysis.

[47] Wu ML (2013). Structural equation modeling-tips for practical application.

[48] Hayes AF (2013). Introduction to mediation, moderation, and conditional process analysis: a regression-based approach.

[49] Sciolla AF, Wilkes MS, Griffin EJ (2019). Adverse childhood experiences in medical students: implications for wellness. Academic Psychiatry.

[50] Monroe SM, Simons AD (1991). Diathesis-stress theories in the context of life stress research: implications for the depressive disorders. Psychol Bull.

[51] Anderson AM, Ross MW, Nyoni JE, McCurdy SA (2015). High prevalence of stigma-related abuse among a sample of men who have sex with men in Tanzania: implications for HIV prevention. AIDS Care.

[52] Risher K, Adams D, Sithole B, Ketende S, Kennedy C, Mnisi Z, Mabusa X, Baral SD (2013). Sexual stigma and discrimination as barriers to seeking appropriate healthcare among men who have sex with men in Swaziland. J Int AIDS Soc.

[53] McLaughlin KA, Sheridan MA, Lambert HK (2014). Childhood adversity and neural development: deprivation and threat as distinct dimensions of early experience. Neurosci Biobehav Rev.

[54] Anda RF, Felitti VJ, Bremner JD, Walker JD, Whitfield C, Perry BD, Dube SR, Giles WH (2006). The enduring effects of abuse and related adverse experiences in childhood – a convergence of evidence from neurobiology and epidemiology. Eur Arch Psy Clin N.

[55] Fuller-Thomson E, Lewis DA (2015). The relationship between early adversities and attention-deficit/hyperactivity disorder. Child Abuse Negl.

[56] Sibley MH, Pelham WE, Molina BSG, Gnagy EM, Waxmonsky JG, Waschbusch DA, Derefinko KJ, Wymbs BT, Garefino AC, Babinski DE (2012). When diagnosing ADHD in young adults emphasize informant reports, DSM items, and impairment. J Consult Clin Psychol.

[57] Harrison AG, Alexander SJ, Armstrong IT (2013). Higher reported levels of depression, stress, and anxiety are associated with increased endorsement of ADHD symptoms by postsecondary students. Can J Sch Psychol Sex Orientat Gend Divers.

[58] Bonanno GA (2004). Loss, trauma, and human resilience: have we underestimated the human capacity to thrive after extremely aversive events?. Am Psychol.

